# Supplementary figures and images for: OXSR1 inhibits inflammasome activation by limiting potassium efflux during mycobacterial infection
Source: Life Sci Alliance. 2022 May 11;5(9):e202201476. doi: 10.26508/lsa.202201476 (PMC9107790; doi:10.26508/lsa.202201476)

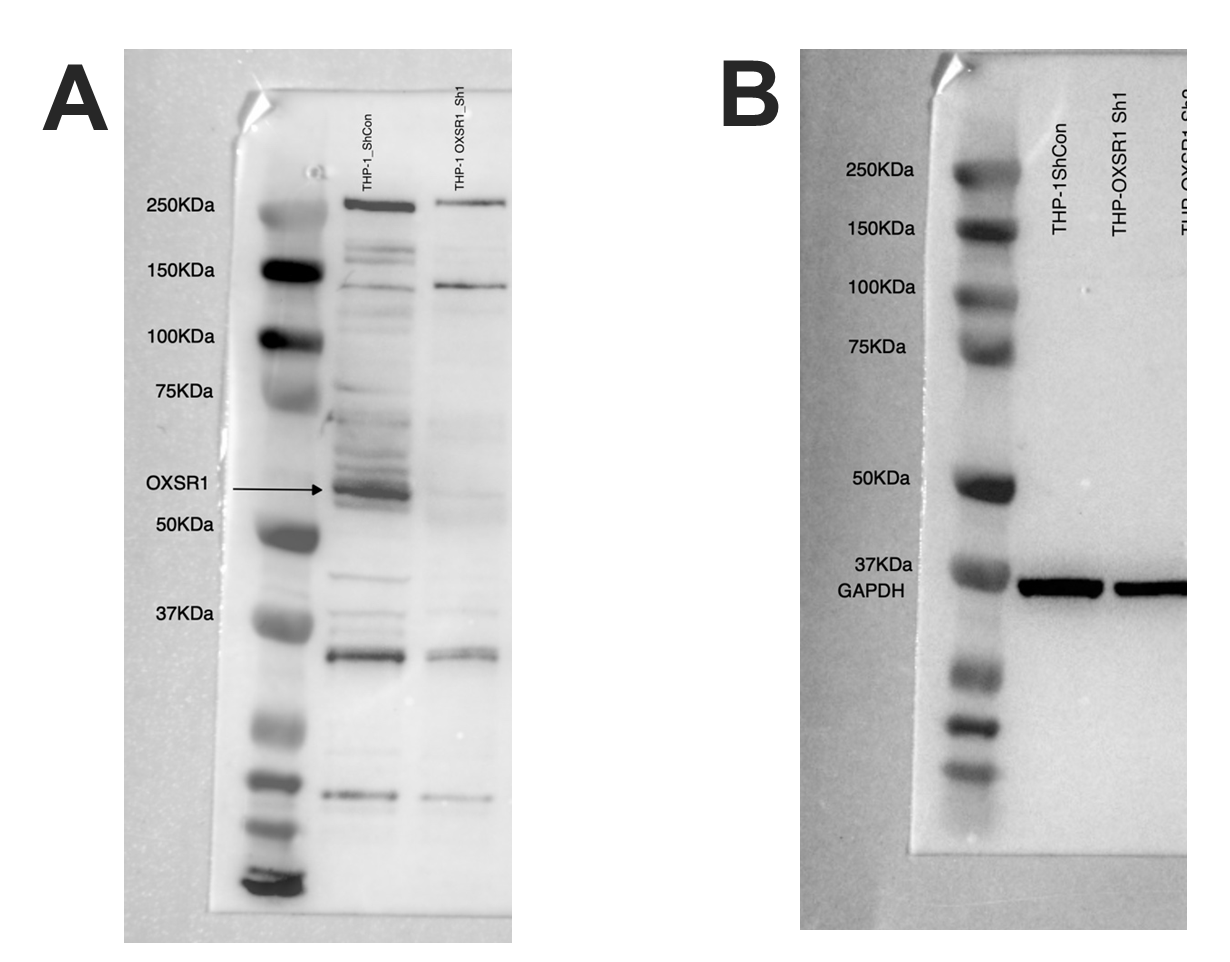

Supplement: Supplementary file 1 [file LSA-2022-01476_SdataF3.tif]
